# Data on cardiac and vascular functionality in *ex vivo* and *in vivo* models following acute administration of trimethylamine N-oxide

**DOI:** 10.1016/j.dib.2023.108890

**Published:** 2023-01-11

**Authors:** Melita Videja, Reinis Vilskersts, Eduards Sevostjanovs, Edgars Liepinsh, Maija Dambrova

**Affiliations:** a Latvian Institute of Organic Synthesis, Aizkraukles street 21, LV-1006, Riga, Latvia; b Faculty of Pharmacy, Riga Stradiņš University, Dzirciema street 16, LV-1007, Riga, Latvia

**Keywords:** Trimethylamine N-oxide, Cardiovascular functionality, Myocardial infarction, Endothelial function, Energy substrate metabolism

## Abstract

This dataset describes in detail the outcomes of acute trimethylamine N-oxide (TMAO) administration on cardiac, vascular and mitochondrial functionality in *ex vivo* and *in vivo* models.

The accumulation of TMAO in target tissues was assessed after performing heart perfusion or by incubating aortic tissue in a solution containing TMAO. To evaluate the impact of TMAO on mitochondrial function, the aortic rings and heart homogenates of Wistar rats were incubated in a solution containing [9,10-^3^H] palmitate (5 µCi/ml) or D-[U-^14^C] glucose (0.625 µCi/ml) in the presence or absence of TMAO with subsequent measurement of substrate oxidation and uptake. The effects of TMAO on the vascular reactivity of isolated conductance and resistance vessels were tested by measuring their response to acetylcholine and sodium nitroprusside. The impact of elevated TMAO levels on cardiac function and infarct size caused by ischemia-reperfusion injury was evaluated in Langendorff perfused heart model. Normal and forced heart functioning was analyzed by echocardiography in CD-1 mouse acute cardiac stress model induced by isoproterenol (10 µg/mouse) upon single and 7 repeated daily administrations of TMAO (120 mg/kg).

The data presented in the manuscript provide valuable information on measurements performed under conditions of acutely elevated TMAO levels in experimental models of cardiac and vascular function and energy metabolism. Furthermore, the data have high reuse potential as they could be applied in the planning of future *in vitro, ex vivo*, and *in vivo* studies addressing the molecular mechanisms targeted by elevated levels of TMAO.

**Specifications Table**

**Table:** 

Subject	Cardiology and Cardiovascular Medicine
Specific subject area	The outcomes after elevated trimethylamine N-oxide (TMAO) levels in *ex vivo* and *in vivo* models of cardiovascular diseases.
Type of data	Figures and table of analyzed data
How the data were acquired	The data were acquired using following methods: • Ultra-performance liquid chromatography-tandem mass spectrometry (UPLC-MS/MS); • In-vitro radiolabeled substrate oxidation followed by scintillation counting; • Assessment of vascular reactivity using isolated organ bath; • Wire myography; • Ex-vivo Langendorff isolated heart; • Echocardiography.
Data format	Raw and analyzed data
Description of data collection	For acute *ex vivo* experiments, samples were obtained from male Wistar rats immediately after euthanasia and incubated in buffer solution containing 100 µM TMAO. For *ex vivo* isolated heart experiments, the perfusion buffer contained 1 mM TMAO. The effects of TMAO on cardiac function *in vivo* were assessed after single or 7-day administration of TMAO in CD-1 mice at a dose of 120 mg/kg.
Data source location	Institution: Latvian Institute of Organic Synthesis City/Town/Region: Riga Country: Latvia Latitude and longitude: 56.976412171042625, 24.191287962011153
Data accessibility	Repository name: Mendeley Data Data identification number: https://doi.org/10.17632/tv72ryssjg.1 Direct URL to data: https://data.mendeley.com/datasets/tv72ryssjg[1]

## Value of the Data

• For more than a decade, TMAO has been studied as a diet- and microbiota-derived metabolite involved in the pathogenesis of cardiometabolic diseases. The data in the manuscript provide useful information on the effects induced by acute TMAO administration in various experimental models for cardiac and vascular functionality.
• These data will be useful for researchers interested in studying the role of TMAO in the development of cardiovascular and metabolic diseases and will help in planning future studies and choosing the experimental conditions for *ex vivo* and *in vivo* experiments.
• The data presented might be reused for comparative studies targeting the acute and chronic effects of increased TMAO concentrations on energy substrate metabolism, cardiac functionality, and vascular reactivity.

## 1 Objective

Trimethylamine N-oxide (TMAO) has previously been associated with a higher risk of cardiovascular and metabolic diseases. Data from the preclinical and clinical setting suggest that it could be a causal factor for the aforementioned diseases [2], however the evidence background is still conflicting. To better understand the role of acutely elevated TMAO level in the pathogenesis of cardiometabolic conditions, a series of experiments were conducted in *ex vivo* and *in vivo* models of cardiac, vascular, and metabolic function. The dataset described in the manuscript expands the knowledge base and provides novel information on the effects of TMAO on the factors affecting disease development.

## 2 Data Description

**Fig. 1:**
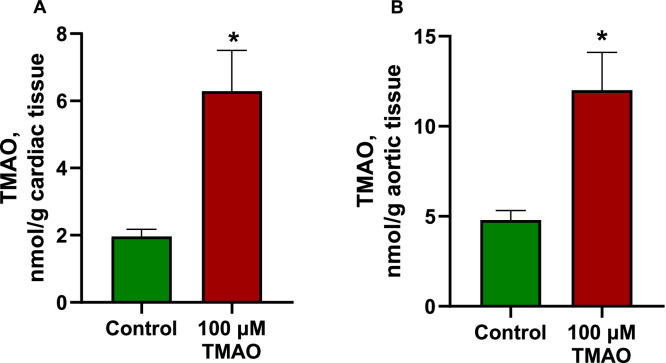
TMAO content in the heart (A) after 1 hour of perfusion and in aortic tissues (B) after 1 hour of incubation in Krebs-Henseleit buffer solution with or without the addition of 100 µM TMAO. The addition of 100 µM TMAO to the buffer solution increased the content of TMAO in cardiac tissue by three and in the aortic rings by two points five times. Data are shown as the mean±SEM of five experiments. * p<0.05 unpaired Student's t test.

The effects of increased concentrations of trimethylamine N-oxide (TMAO) in incubation buffer on tissue accumulation of TMAO were studied (Fig. 1). For this, samples of male Wistar rat hearts were perfused, and aortic rings were immersed in Krebs-Henseleit (K-H) buffer solution with or without 100 µM TMAO. After 1 hour of perfusion or incubation, the samples were further prepared for UPLC/MS/MS analysis to assess the tissue content of TMAO. After perfusion with a buffer solution containing TMAO, the TMAO content in cardiac tissue increased 3 times (from 2.0±0.2 to 6.3±1.2 nmol/mg tissue) and ∼2.5 times in aortic tissue after incubation (from 4.8±0.5 to 12.0±1.2 nmol/mg tissue).

**Fig. 2:**
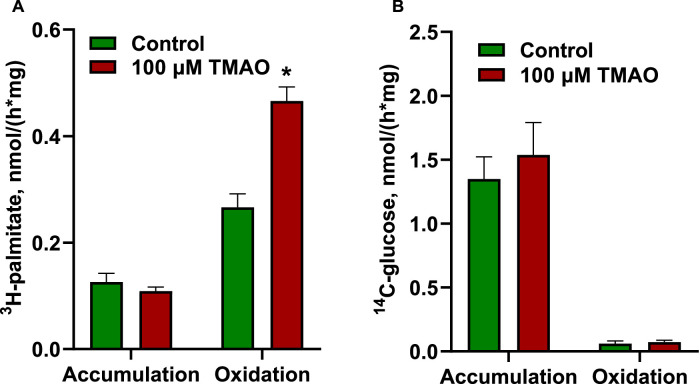
Effects of 100 µM TMAO on the accumulation and oxidation of ^3^H-palmitate (A) and ^14^C-glucose (B) in aortic tissues. Incubation of rat aortic rings with 100 µM TMAO increased ^3^H-palmitate oxidation but did not influence ^3^H-palmitate accumulation or ^14^C-glucose accumulation and oxidation. Data are shown as the mean±SEM of five to six experiments. * p<0.05 unpaired Student's t test.

**Fig. 3:**
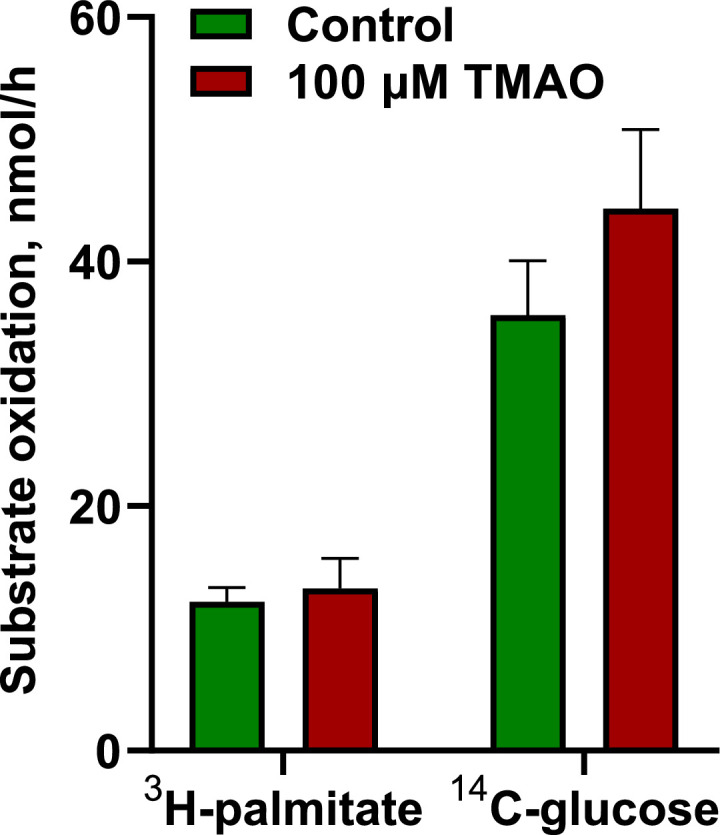
Effects of 100 µM TMAO on the oxidation of ^14^C-glucose and ^3^H-palmitate in heart muscle homogenates. Incubating heart homogenate with 100 µM TMAO did not affect ^14^C-glucose and ^3^H-palmitate oxidation. Data are shown as the mean±SEM of five experiments.

Furthermore, the acute impact of 100 µM TMAO on energy substrate metabolism was assessed by measuring the oxidation of radiolabeled glucose and palmitate and their accumulation in rat aortic rings (Fig. 2). Incubating aortic rings with 100 µM TMAO increased palmitate oxidation nearly two-fold (from 0.26±0.03 to 0.46±0.03 nmol/(h × mg) ^3^H-palmitate); however, glucose metabolism was not affected. For cardiac tissue, the reaction was performed in tissue homogenates; therefore, only the oxidation of radiolabeled glucose and palmitate was evaluated (Fig. 3). TMAO did not affect energy substrate oxidation in heart homogenates.

**Fig. 4:**
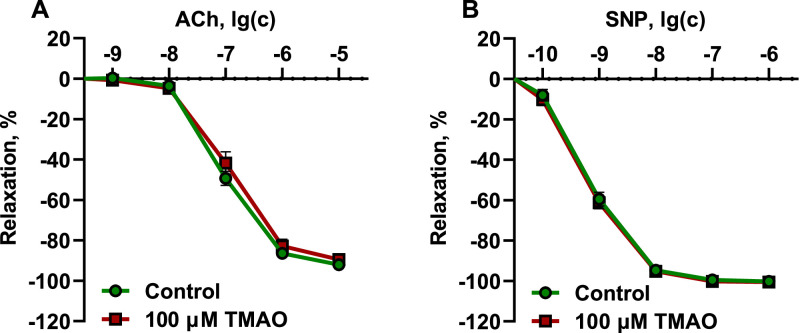
Effects of 100 µM TMAO on endothelium-dependent (A) and endothelium-independent (B) relaxation in aortic rings. Incubation of the aortic rings for 1 h with 100 µM TMAO did not affect endothelium-dependent and endothelium-independent relaxation. The data are shown as the mean±SEM of twelve aortic rings.

**Fig. 5:**
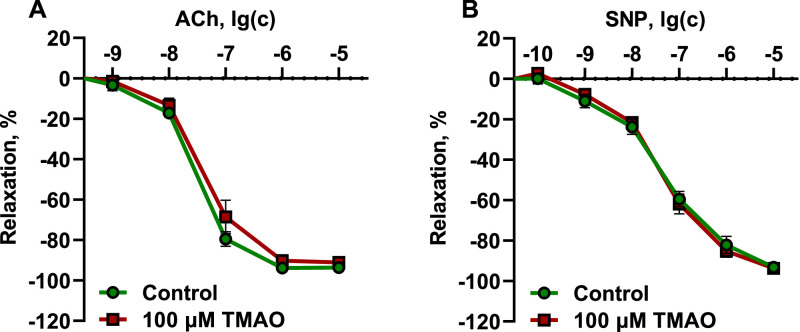
Effects of 100 µM TMAO on endothelium-dependent (A) and endothelium-independent (B) relaxation in mesenteric artery rings. Incubating the mesenteric artery rings for 1 h with 100 µM TMAO did not affect endothelium-dependent and endothelium-independent relaxation. The data are shown as the mean±SEM of seven to eight mesenteric artery rings.

Next, the potency of TMAO to affect the reactivity of conductance and resistance vessels was evaluated. Isolated organ bath experiments were conducted in rat aortic rings submerged in a K-H buffer solution with or without 100 µM TMAO for 1 hour to assess the response to acetylcholine (endothelium-dependent relaxation) and sodium nitroprusside (endothelium-independent relaxation) (Fig. 4). A similar procedure was performed on wire myograph with mesenteric arteries (Fig. 5) to assess whether resistance vessels were affected by TMAO in a similar way as conductance vessels. Incubating the rings of both the aorta and mesenteric artery with 100 µM TMAO did not alter endothelium-dependent or independent relaxation.

**Fig. 6:**
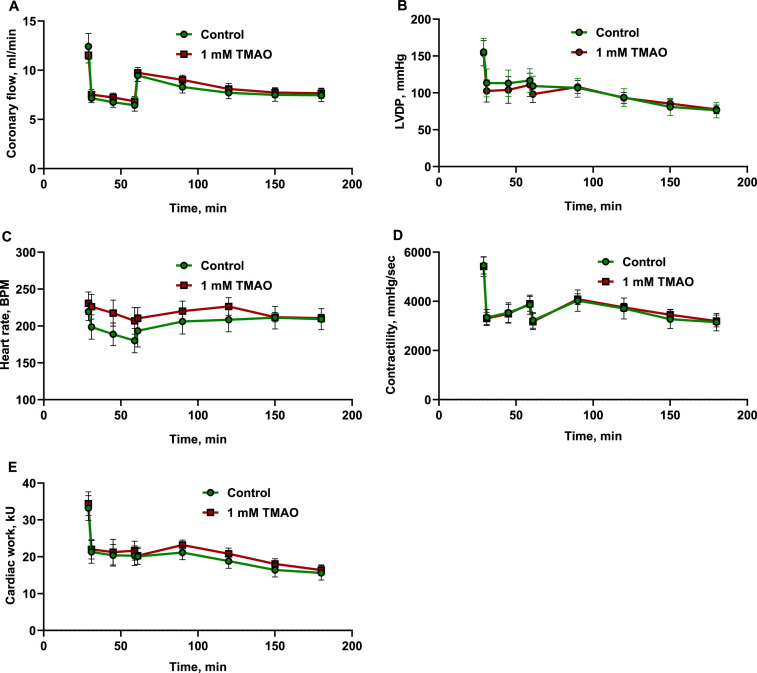
Effects of 1 mM TMAO in the perfusion buffer solution on functional parameters (coronary flow (A), LVDP (B), heart rate (C), contractility (D), and cardiac work (E)) of the isolated rat heart before and during 30 min of occlusion with 120 min of reperfusion. Perfusion of the isolated rat heart with buffer solution containing 1 mM TMAO did not influence heart function during ischemia‒reperfusion. The data are shown as the mean±SEM of six hearts.

**Fig. 7:**
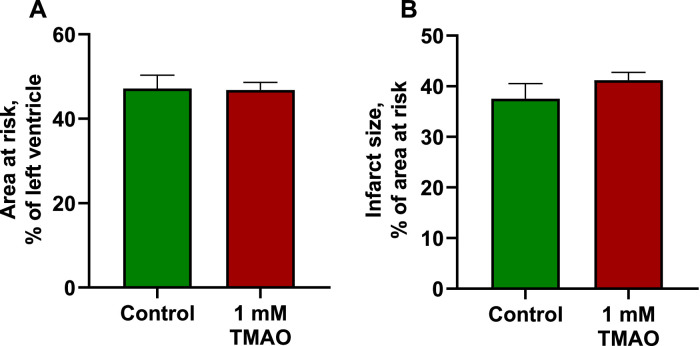
Effects of 1 mM TMAO on the size of myocardial infarction after 30 min of performing occlusion with the left anterior descending coronary artery and then 120 min of reperfusion. Hearts from both groups showed a similar area at risk in the left ventricle (A). Perfusion of the isolated rat heart with a KH buffer solution containing 1 mM TMAO did not influence the size of the myocardial infarction (B). The data are shown as the mean±SEM of six hearts.

In addition, the effects of elevated TMAO concentrations on cardiac function were assessed. First, cardiac functional parameters were tested in a Langendorff isolated rat heart model in the presence of 1 mM TMAO in K-H perfusion buffer (Table 1). Next, the effects of elevated TMAO concentrations in the perfusion buffer (1 mM) on overall cardiac functionality during ischemia‒reperfusion were evaluated (Fig. 6). Perfusion of isolated rat hearts with 1 mM TMAO caused no effect on heart function (heart rate, coronary blood flow, contractility, left ventricle developed pressure and cardiac work) at the baseline or during ischemia‒reperfusion. After this, the size of myocardial infarction was compared in both groups (Fig. 7). After 30 min of left anterior descending artery occlusion and then 2 h of reperfusion, the necrosis zone was nearly identical (∼40% of the risk zone) in both groups.

**Table 1:** Effects of 1 mM TMAO on heart function. Perfusion of the isolated rat heart with K-H buffer solution containing 1 mM TMAO did not alter functional parameters of the heart.

	Control	1 mM TMAO
Coronary Flow	12.4±1.3	11.5±0.8
LVDP	155±19	153±17
Heart rate	219±12	230±15
Contractility	5458±347	5419±399
Cardiac Work	33±4	34±3

The results are shown as the mean±SEM of six hearts.

**Fig. 8:**
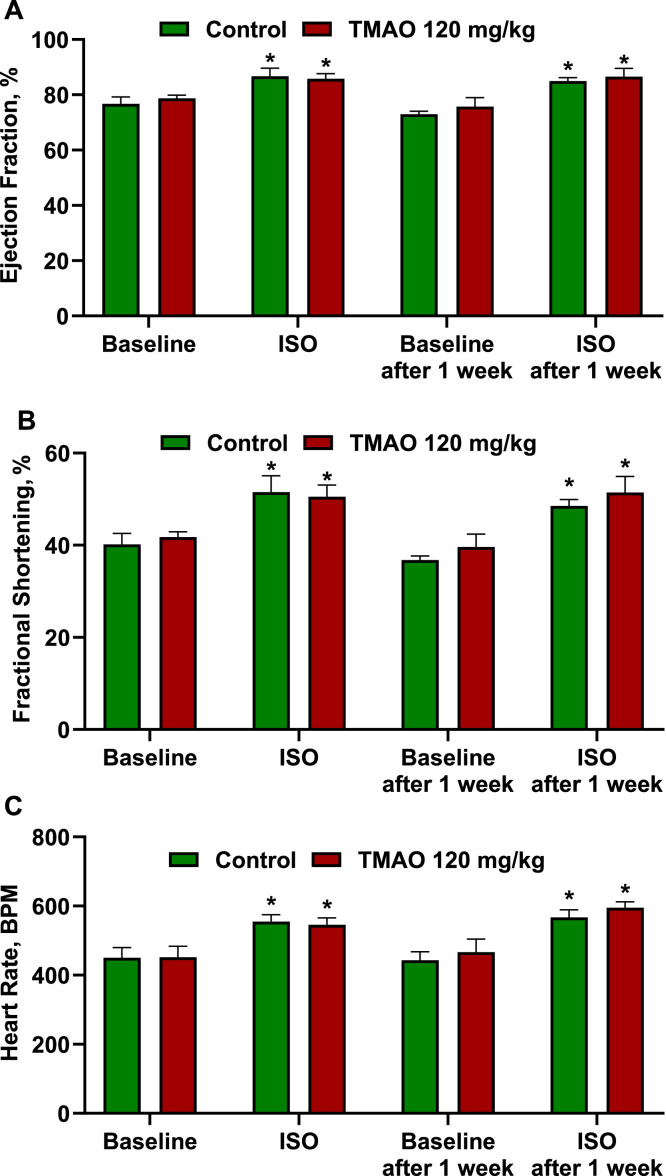
Effects of single and 7-day administration of TMAO at a dose of 120 mg/kg on left ventricular ejection fraction (A), fractional shortening (B), and heart rate (C). Single and 7-day administration of TMAO did not change the functional parameters of the heart. The results are shown as the mean±SEM of six animals.

Last, the effects of single and 7-day administration of TMAO at a dose of 120 mg/kg in CD-1 mice on the cardiac response to isoproterenol-induced acute cardiac stress in mice were tested (Fig. 8). Administration of TMAO at a 120 mg/kg dose caused no effect on the baseline systolic function of the left ventricle. The ejection fractions in the control and TMAO-treated groups were 77±3% and 79±1%, respectively. Treating animals with isoproterenol (10 µg/mouse) significantly increased left ventricular ejection fraction, fractional shortening, and heart rate; however, neither single administration nor 7-day administration of TMAO at a dose of 120 mg/kg showed any effect on these parameters.

## 3 Experimental Design, Materials and Methods

### 3.1 Chemicals

TMAO dihydrate was obtained from Alfa Aesar (Kandel, Germany). Sodium pentobarbital (Dorminal) solution was purchased from Alfasan (Woerden, Holland). Heparin sodium was purchased from Panpharma (Fougeres, France). Acetonitrile and methanol (HPLC grade) were purchased from Merck (Darmstadt, Germany), and 98% formic acid (LC/MS grade) was obtained from Fluka (Buchs, Switzerland). [9,10-^3^H]-Palmitate (5 µCi/ml) or D-[U-^14^C]-glucose (0.625 µCi/ml) was purchased from Biotrend (Zürich, Switzerland). ATP was purchased from TCI (Antwerp, Belgium). Isoflurane was purchased from Chemical Point (Deisenhofen, Germany). All other reagents were purchased from Sigma‒Aldrich (Schnelldorf, Germany).

### 3.2 Animals and treatment

Thirty male Wistar rats weighing 200–250 g were obtained from the Laboratory Animal Centre, University of Tartu (Tartu, Estonia). Twelve male CD-1 mice at the age of 6-8 weeks weighing 25-30 g were obtained from the Laboratory Animal Centre, University of Tartu (Tartu, Estonia). All experimental animals were housed under standard conditions (21-23°C, 12-hour light/dark cycle, relative humidity 45-65%) with unlimited access to food (R70 diet, Lactamin AB, Kimstad, Sweden) and water. Rats (n=12) were used to determine the levels of TMAO in vascular and myocardial tissue. Six rats were used to assess the effects of TMAO on energy substrate oxidation and vascular reactivity after incubation in TMAO-containing (100 µM) buffer solution, and 12 rats were used in the isolated heart experiments to assess the functionality of the heart and the size of myocardial infarction after perfusion with 1 mM TMAO. Mice (n=12) were used to study the effects of TMAO administration on heart functionality and the response to adrenergic stimulation.

To obtain cardiac and vascular tissue for *ex vivo* experiments, the rats were anesthetized with an intraperitoneal injection of sodium pentobarbital (60 mg·kg−1) and heparin (1000 IU·kg−1). After the onset of anesthesia, the thorax was opened, and the heart and thoracic aorta were removed and placed into ice-cold Krebs-Henseleit (K-H) buffer solution (composition (in mmol/L): NaCl 118, CaCl_2_ 2.5, MgCl_2_ 1.64, NaHCO_3_ 24.88, KH_2_PO_4_ 1.18, glucose 10.0, and EDTA 0.05; pH 7.4 at 37°C) until the tissue was further processed.

### 3.3 Determination of TMAO concentrations

Rat hearts were perfused, and aortic rings from each experimental animal were immersed in K-H buffer solution with or without the addition of TMAO (100 µM final concentration). After 1 hour of perfusion or incubation, the tissue samples were washed to eliminate the residues of TMAO-containing buffer solution and further homogenized with water in an OMNI Bead Ruptor 24 (Camlab, UK) at a w/v ratio of 1:10. The homogenate was then centrifuged at 20000 × g for 10 min at 4°C. The supernatants were collected and stored frozen (− 80°C) until further analysis.

The TMAO concentrations in the aorta and heart homogenate samples were measured by ultra-performance liquid chromatography-tandem mass spectrometry (UPLC/MS/MS) using a positive ion electrospray, as previously described [3,4]. In brief, the sample preparation was performed by deproteinization with an acetonitrile/methanol mixture (3:1, v/v) and centrifugation at 13000 × g for 10 min. The supernatant was then transferred to UPLC vials and analyzed using UPLC/MS/MS. Data acquisition and further processing were carried out in MassLynx 4.1. software with a QuanLynx 4.1. module (Waters, Milford, USA).

### 3.4 Energy substrate oxidation

To assess the energy substrate oxidation rate, cardiac tissues were minced with scissors and homogenized with a Turrax homogenizer (IKA, Staufen, Germany). Samples were prepared at a ratio of 1:10 w/v in Isolation Buffer A (composition (in mmol/L): KCl 180, Tris-base 10, EDTA 0.5). Heart homogenates were centrifuged at 1000 × g for 5 min, and the supernatant was used to assess fatty acid and glucose oxidation. The aortic rings remained intact. The reaction mix for fatty acid oxidation contained 1 mM NAD, 5 mM ATP, 100 µM CoA, 1 mM malate, 700 µM L-carnitine, and [9,10-^3^H] palmitate (specific activity, 5 µCi/ml) in K-H buffer solution with or without 100 µM TMAO. The glucose oxidation reaction mix consisted of 1 mM NAD, 5 mM ATP, 100 µM CoA and D-[U-^14^C] glucose (specific activity, 0.625 µCi/ml) in K-H buffer solution with or without 100 µM TMAO.

Glucose and palmitate oxidation rates were determined as described previously [5]. Briefly, glucose oxidation was assessed by measuring the ^14^CO_2_ released from the metabolism of D-[U-^14^C] glucose. Palmitate oxidation was determined by measuring the ^3^H_2_O released from [9,10-^3^H] palmitate. Glucose and palmitate uptake in the aorta was calculated from the amount of radiolabeled substrates oxidized and the amount found in aortic tissues when the reaction ended.

### 3.5 Effects of TMAO on vascular reactivity of conductance and resistance vessels

Vascular reactivity of aortic rings was assessed as described previously [6]. In brief, the excised thoracic aorta was immersed in ice-cold K-H buffer solution and the surrounding tissue were removed. The vessels were cut into 3- to 4-mm-long rings that were suspended between two stainless steel hooks in a 10 mL organ bath filled with K-H buffer solution saturated with 95% O_2_ and 5% CO_2_, and four parallel samples were prepared from the same animal. The aortic rings were stretched to a resting tension of 2 g and equilibrated to the new conditions for 60 min. During this adaptation, the incubation buffer solution was changed every 15 min. The maximal contraction force of each ring was determined by adding 60 mM potassium chloride. After washing, TMAO at a concentration of 100 µM was added to half of the aortic rings and incubated for 1 h. Aortic rings were then washed once more with buffer solution and precontracted with phenylephrine to 70%-80% of maximal contraction. Endothelium-dependent relaxation was assessed by adding cumulative concentrations of acetylcholine (10^−9^ to 10^−5^ mol/L). Endothelium-independent relaxation was assessed by adding cumulative concentrations of SNP (10^−10^ to 10^−5^ mol/L). The relaxation of the aortic rings in response to acetylcholine or SNP was expressed as a percentage of the phenylephrine-induced constriction.

Vascular reactivity in mesenteric artery rings was assessed as described previously [7] with modifications. The intestine with the mesenteric arcade attached was excised and transferred to ice-cold physiological salt solution (PSS) with the following composition (mmol/L): NaCl 130, KCl 4.7, MgSO_4_ 1.17, KH_2_PO_4_ 1.18, NaHCO_3_ 14.9, glucose 5.5, and EDTA 0.026. Second-order mesenteric arteries were cleaned and dissected of adjoining fat and connective tissues. The arteries were cut into ring segments 2 mm in length, and four parallel samples were prepared from the same animal. Each segment was mounted in a Multi-Myograph System (Danish Myograph Technology, Aarhus, Denmark) in PSS saturated with a gas mixture of 95% O_2_ and 5% CO_2_. Further assessment of endothelium-dependent and endothelium-independent function after incubation with 100 µM TMAO was performed in a similar manner as for the aorta.

### 3.6 Experimental heart infarction *ex vivo*

The infarction was performed according to the Langendorff technique as described previously [8], with some modifications. For the infarction studies, the hearts were perfused with K-H buffer solution with or without 1 mM TMAO at a constant perfusion pressure of 60 mmHg. Heart rate, left ventricular end-diastolic pressure, and left ventricular developed pressure were recorded continuously. Coronary flow was measured using an ultrasound flow detector (HSE) and PowerLab systems from ADInstruments. The isolated rat hearts were left to adapt for 30 min, and then the left anterior descending coronary artery (LAD) was occluded for 30 min, followed by 120 min of reperfusion. Further analysis was performed as described by Liepinsh et al. [9]. In brief, LAD was reoccluded and perfused with 0.1% methylene blue. Afterward, the heart was transversely cut into 2‐mm‐thick slices, treated with triphenyltetrazolium chloride (TTC) and photographed. Computerized planimetric analysis was carried out using Image‐Pro Plus v6.3 software (Media Cybernetics Inc., Rockville, MD, USA) to determine the area at risk (AR) and the area of necrosis (AN). Each area was then expressed as a percentage of the total left ventricle area. The infarct size (IS) was then calculated as a percentage of the risk area according to the formula IS = AN/AR × 100%.

### 3.7 Isoproterenol-induced cardiac stress model

The isoproterenol-induced acute cardiac stress model was established as previously described [10] with some modifications. Before the study, experimental animals were randomly divided into two experimental groups (n=6) and weighed. Mice from both groups were anesthetized using 5% isoflurane dissolved in 100% oxygen. After the onset of anesthesia, the concentration of isoflurane was decreased to 2.5%, the experimental animals were placed in a decubitus position, and the chest was shaved. M-mode tracings of the left ventricle were recorded at the papillary muscle level using an iE33 ultrasonograph equipped with a linear L15-7io transducer (Philips Health care, Andover, USA). Afterward, the mice from the first experimental group received isoproterenol at a dose of 10 µg/mouse, but the animals from the second group received isoproterenol and TMAO at doses of 10 µg/mouse and 120 mg/kg, respectively. After 30 min, the experimental animals were anesthetized with isoflurane once more to record the cardiac response to acute cardiac stress and the impact of TMAO on the inotropic and chronotropic effects. For the next seven days, the mice in the second group received TMAO together with drinking water at a dose of 120 mg/kg, while the animals from the first group received pure drinking water. After one week of treatment, the experimental animals were anesthetized, and echocardiography was performed before and after administration of isoproterenol or a combination of isoproterenol and TMAO as described before.

### 3.8 Statistical methods

All data are represented as the mean ± standard error of the mean (SEM). For statistical analysis, Student's t test or one-way ANOVA with Tukey's post-test were used. A two-sided p value less than 0.05 was considered statistically significant. Statistical calculations were performed using Prism software (GraphPad, San Diego, California).

## Ethics Statements

The experimental procedures described here were performed in accordance with the EU Directive 2010/63/EU for animal experiments and local laws and policies. All procedures were approved by the Latvian Animal Protection Ethical Committee of the Food and Veterinary Service, Riga, Latvia. *Ex vivo* experiments were performed in compliance with ethical approval Nr. 82; isoproterenol-induced cardiac stress model was performed in compliance with ethical approval Nr. 84. All studies involving animals are reported in accordance with the ARRIVE guidelines [11].

## CRediT authorship contribution statement

**Melita Videja:** Conceptualization, Data curation, Formal analysis, Visualization, Writing – original draft, Funding acquisition. **Reinis Vilskersts:** Conceptualization, Methodology, Data curation, Formal analysis, Writing – review & editing. **Eduards Sevostjanovs:** Data curation. **Edgars Liepinsh:** Conceptualization, Supervision. **Maija Dambrova:** Conceptualization, Supervision, Writing – review & editing, Funding acquisition.

## Declaration of Competing Interest

The authors declare that they have no known competing financial interests or personal relationships that could have appeared to influence the work reported in this paper.

## Data Availability

Data on cardiac and vascular functionality in ex vivo and in vivo models following acute administration of trimethylamine N-oxide (Original data) (Mendeley Data).
